# Tasquinimod triggers an early change in the polarization of tumor associated macrophages in the tumor microenvironment

**DOI:** 10.1186/s40425-015-0098-5

**Published:** 2015-12-15

**Authors:** Anders Olsson, Jessica Nakhlé, Anette Sundstedt, Pascale Plas, Anne-Laure Bauchet, Valérie Pierron, Luce Bruetschy, Adnan Deronic, Marie Törngren, David Liberg, Fabien Schmidlin, Tomas Leanderson

**Affiliations:** Active Biotech AB, Lund, Sweden; Global Drug Discovery Department, IPSEN Innovation, 91966 Les Ulis, France; Immunology Group, Lund University, Lund, Sweden

**Keywords:** TME, TAMs, Macrophage polarization, CD206, IL-12, Tasquinimod, Immune therapy

## Abstract

**Background:**

Tasquinimod (a quinoline-3-carboxyamide) is a small molecule immunotherapy with demonstrated effects on the tumor microenvironment (TME) involving immunomodulation, anti-angiogenesis and inhibition of metastasis. A target molecule of tasquinimod is the inflammatory protein S100A9 which has been shown to affect the accumulation and function of suppressive myeloid cell subsets in tumors. Given the major impact of myeloid cells to the tumor microenvironment, manipulation of this cell compartment is a desirable goal in cancer therapeutics.

**Methods:**

To understand the consequences of tasquinimod treatment on the TME, we evaluated early treatment effects in tumor infiltrating myeloid cells. Cellular phenotypes were studied by flow cytometry while gene expression both in tumor tissue and in isolated CD11b^+^ cells or tumor cells were measured by real time-PCR. Effects on angiogenesis were monitored by changes in CD31 levels and by gene expression in tumor tissue. Effects on cytokine levels in tumor tissue and serum were determined by multiplex analysis.

**Results:**

The MC38-C215 colon carcinoma tumors showed a substantial infiltration of primarily myeloid cells that were dominated by Ly6C^low^F4/80^+^CD206^+^ M2-polarized tumor associated macrophages (TAMs), an immuno-suppressive and pro-angiogenic cell population. Here, we show that tasquinimod treatment induces an anti-tumor effect which is subsequent to a reduction in tumor infiltrating CD206^+^ M2 macrophages and a simultaneous increase in M1 macrophages expressing MHC class II and CD86. The tasquinimod-induced changes in TAM polarization were evident within 24 h of exposure, emphasizing the ability of tasquinimod to rapidly reprogram the tumor microenvironment. This change in the tumor associated myeloid compartment preceded an increased IL12-production within the tumor and a decrease in tumor neovascularization. The switch in TAM polarization by tasquinimod was confirmed in the 4T1 breast cancer model where tasquinimod also reduce lung metastasis development.

**Conclusion:**

Our data show that tasquinimod affects tumor infiltrating myeloid cells early after exposure, leading to a change in phenotype from pro-angiogenic and immunosuppressive M2-like TAMs to pro-inflammatory M1-like macrophages. These changes are consistent with the effects of tasquinimod seen on tumor vascularization, immune suppression and metastasis giving further insights to the anti-tumor mechanism of action of tasquinimod.

**Electronic supplementary material:**

The online version of this article (doi:10.1186/s40425-015-0098-5) contains supplementary material, which is available to authorized users.

## Background

During the last decades it has become evident that pharmacological targeting of the tumor microenvironment (TME) is of major importance for a successful clinical outcome of anti-tumor therapies. Consequently, a number of different therapeutic strategies are being developed to target the cells and molecules of the TME [1–5]. The TME contains fibroblasts and endothelial cells but also infiltrating lymphocytes and regulatory myeloid cells such as myeloid derived suppressor cells (MDSCs) and tumor associated macrophages (TAMs). These multiple stromal cells constitute the primary tumorigenic niche and their interactions are critical for tumor growth and metastasis [6].

Macrophages are plastic cells and can be polarized towards (i) classically activated, pro-inflammatory M1 macrophages or (ii) alternatively activated, anti-inflammatory and immune-suppressive M2 macrophages [7, 8]. M2 macrophages inhibit T cell activation by e.g. depletion of L-arginine via arginase-1 (Arg-1) [9, 10], express the mannose receptor C type 1 (Msr1; also called CD206) and promote angiogenesis by expressing Tie-2 and by producing the pro-angiogenic factor VEGF-A [11–13]. M1 macrophages on the other hand produce anti-angiogenic cytokines such as IL-12 [14, 15] and promote immune-mediated anti-tumor activity via production of inducible nitric oxide synthase (iNOS) and by high expression of MHC class II molecules [8]. Although TAMs are usually depicted as M2 macrophages, both forms can be found in the TME. Polarization seems to depend on the nature of the TME where the localization, type and origin of the tumor, hypoxia, other infiltrating cells, and tumor produced factors is highly associated with the phenotype and function of the infiltrating TAMs [16]. Signaling through nuclear factor kappa B (NF-κB) is considered of major importance for macrophage polarization [10]. For instance, it has been shown that sustained nuclear expression of p50 NFκB homodimer results in a M2 phenotype [17], while by targeting IKKβ and thereby inhibiting NF-κB activity, M2 TAMs could be converted into macrophages of the M1 phenotype [18, 19].

Tasquinimod (ABR-215050; a quinoline-3-carboxyamide) is a small-molecule compound that has shown immunomodulatory [20, 21], anti-angiogenic [22, 23] and anti-metastatic [24] properties in several experimental tumor models. In a phase II clinical study, tasquinimod demonstrated improved progression free survival compared to placebo in men with minimally symptomatic metastatic castration-resistant prostate cancer [25]. In a randomized placebo-controlled phase III pivotal clinical study, tasquinimod reduced the risk of radiographic cancer progression or death compared to placebo (rPFS, HR = 0.69, 95 % CI: 0.60–0.80) in patients with metastatic castration-resistant prostate cancer who had not received chemotherapy, but tasquinimod did not extend overall survival (HR = 1.097, 95 % CI: 0.938–1.282) [26]. Tasquinimod interacts with and blocks the function of two proteins that both are important for signaling in the tumor microenvironment. One is the inflammatory protein S100A9 [20, 27] that can be secreted as a Damage Associated Molecular Pattern (DAMP) to interact with receptors such as TLR4, RAGE and CD147 (EMMPRIN) on myeloid and other cells. S100A9 has been demonstrated to affect the accumulation and function of CD11b^+^Gr1^+^ regulatory myeloid cells [28, 29], and has multiple functions in macrophage subsets [30]. Another molecular target for tasquinimod is histone-deacetylase-4 (HDAC4) that is involved in HIF1α-signaling. Binding of tasquinimod to HDAC4 prevents it from forming an active complex with NCoR/HDAC3 and inhibits HDAC4 client transcription factors such as HIF-1α [31].

Tasquinimod treatment leads to changes in the number and frequency of tumor-infiltrating regulatory myeloid cells and reduces the immune suppressive potential of the TME [21, 24]. In this study, we extended the analysis of consequences of tasquinimod treatment on the TME looking at early changes in myeloid cells after treatment. Surprisingly, tasquinimod treatment altered the TAM population in the tumor as early as 24 h after initiating treatment in the MC38-C215 colon tumor model. Increased levels of IL-12 could be detected within the tumor after 5 days which preceded changes in the vasculature of the tumor. Macrophages isolated from tumors at the end of the experiment showed changes in both gene expression and function that reflected a shift from M2 to M1 macrophages. This phenotypic switch was confirmed in the 4T1 mammary tumor model where it also preceeded a reduction in lung metastasis. Thus, tasquinimod treatment induced a prompt and robust effect on myeloid cells in tumors. Given the major contribution of tumor-infiltrating myeloid cell populations to angiogenesis, immune suppression and metastatic potential of a tumor, these findings provide more insights into the effects of tasquinimod in these processes. This study also highlights the adaptability of the TME by showing the possibility to induce early changes in TAM polarization *in vivo* by inhibition of signals important to maintain the pro-tumoral functions of the TME.

## Results

### Tasquinimod treatment impairs MC38-C215 tumor growth in vivo

**Fig. 1:**
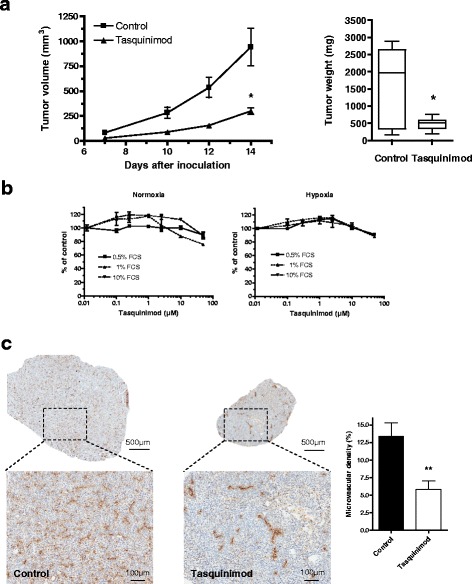
Tasquinimod impairs tumor growth in vivo and reduces the microvascular density in MC38-C215 tumors. **a** Wild type mice were inoculated s.c. with 0.5 × 10^6^ MC38-C215 cells. Treatment with tasquinimod (30 mg/kg ad lib.) was initiated on the day of tumor inoculation and continued throughout the experiment. Tumor volumes (left panel) were measured every 2-3 days from day 7 and the experiment was terminated on day 14 (*p = 0.029; two-way ANOVA, Error bars indicate s.e.m) and tumor weights (right panel) at the end of experiment (*p < 0.05; Mann Whitney). **b** No effect on MC38-C215 proliferation *in vitro.* MC38-C215 cancer cells (5000 cells/well) were seeded in 96 well plates and the cultured cells treated with tasquinimod for 72 h under either normoxic (left panel) or hypoxic (right panel) growth conditions. Viability was determined by MTT assay at the end of incubation. **c** CD31 staining is significantly different between tumors from control and tasquinimod treated animals: Left-control group tumor, right-tasquinimod group tumor. Vascular density determined as percentage of stained area compared to total tumor area (* *p* < 0.05; Mann Whitney, Error bars indicate s.e.m)

Tasquinimod has previously demonstrated potential therapeutic benefit in several xenograft tumor models which has been mainly linked to its anti-angiogenic properties [22]. However, in a recent study in two different syngeneic tumor models, tasquinimod was shown to modulate myeloid cells, leading to a reduction in tumor immunosuppression and increased efficacy of two different immunotherapies [21]. To further study these changes and to investigate the crosstalk between tasquinimod's immunomodulatory and anti-angiogenic properties, experiments were performed in the MC38-C215 tumor model, a variant of the syngenic MC38 colon carcinoma model which has a strong component of TAMs with a predominance of the M2-phenotype [32]. We demonstrated here that tasquinimod treatment led to a significant inhibitory effect on MC38-C215 tumor growth after subcutaneous inoculation of tumor cells (Fig. 1a). In contrast to its effect on MC38-C215 tumor growth *in vivo*, tasquinimod had no direct effect on MC38-C215 cell proliferation *in vitro*, neither under normoxic or hypoxic growth conditions (Fig. 1b). The anti-tumor effect *in vivo* did not correlate with an increased T cell infiltration since only a few CD4 or CD8 positive cells were observed in the MC38-C215 tumors at end-point without significant differences between control and tasquinimod treated tumors (unpublished observations). However, the treated tumors showed a significant reduction in microvessel density as detected by CD31 staining (Fig. 1c). Thus, tasquinimod inhibits growth of MC38-C215 tumors in syngeneic mice *via* an effect that does not correlate to a general inhibition of tumor cell proliferation, nor to an increased T cell infiltration, but that does correlate to a change in vascularization of the tumor.

### Tumor-infiltrating TAMs are functionally skewed from a M2 to a M1 phenotype after tasquinimod treatment

**Fig. 2:**
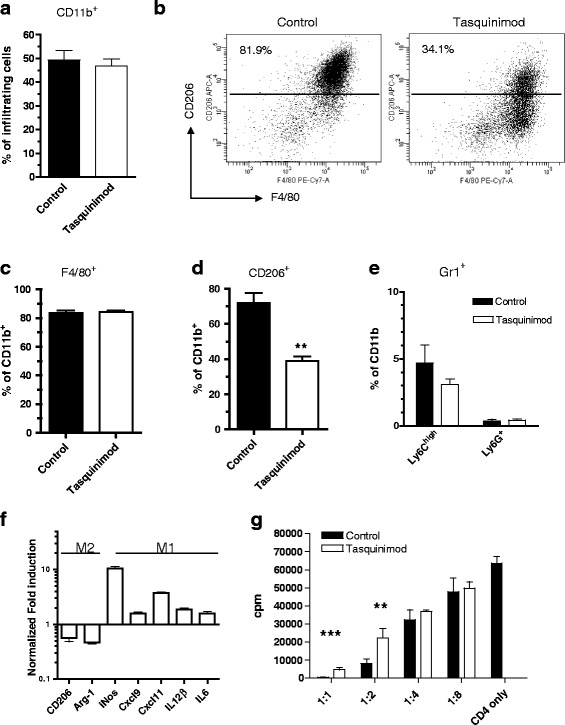
CD11b^+^F4/80^+^ infiltrating cells are functionally skewed from a pro-tumor CD206^+^ M2 phenotype into a CD206^−^ anti-tumor M1 phenotype after tasquinimod treatment. Modulation of myeloid CD11b^+^ subpopulations in MC38 tumors by tasquinimod. **a** Percent CD11b^+^ cells of infiltrating cells, **b** representative FACS plots F4/80^+^CD206^+^ staining of tumor infiltrating CD11b^+^ cells, **c** F4/80^+^ cells as frequency of total CD11b^+^ cells, **d** frequency of CD206^+^ cells in tumors ± tasquinimod treatment (**p < 0.01; two-way ANOVA, Error bars indicate s.e.m) and **e** frequency of Ly6C^high^Ly6G^−^ and Ly6G^+^ cell populations in tumor. **f** Up-regulation of M1 markers and down-regulation of M2 markers in infiltrating CD11b^+^F4/80^+^ after tasquinimod treatment. Tumor infiltrating CD11b^+^F4/80^+^ cells were isolated from MC38-C215 tumors at day 14, FACS-sorted and qRT-PCR was performed on the indicated genes. **g** Infiltrating F4/80^+^ cells are less immune suppressive after tasquinimod treatment. CD4^+^ T cells were stimulated with αCD3 and α CD28 in the presence of F4/80^+^ cells at indicated ratios (F4/80:CD4) and proliferation was measured by ^3^H-thymidine incorporation (**p < 0.01 and ***p < 0.001; *t*-test, Error bars indicate s.e.m)

Tasquinimod has shown effects on myeloid cells in other experimental tumors [21] and given the major contribution of regulatory myeloid cells to tumor angiogenesis [2], we analyzed the consequence of tasquinimod treatment on the myeloid cell compartment in the spleen and in the tumor. The frequency of CD11b-expressing cells in the tumors was not affected by tasquinimod treatment for 14 days (Fig. 2a). The CD11b^+^ compartment in these tumors consisted of more than 80 % of F4/80^+^ cells and the majority of the cells in the untreated tumors also expressed CD206 (Fig. 2b, left panel). F4/80 and CD206 together mark M2-skewed TAMs, that have both immunosuppressive and pro-angiogenic functions. Treatment with tasquinimod changed the CD11b^+^F4/80^+^ population from a CD206^+^ to a mainly CD206^−^ population at the experimental endpoint (day 14) (Fig. 2b-d), indicative of a shift from immunosuppressive M2 to pro-inflammatory M1 macrophages. While F4/80^+^ cells were the dominating population of the tumor infiltrating myeloid cells, the granulocytic Ly6G^+^ and monocytic Ly6C^high^ MDSC subpopulations were only found at low levels in the tumor and were not significantly altered by tasquinimod treatment (Fig. 2e). To further investigate the M2 to M1 switch induced by tasquinimod, a similar analysis was performed in the orthotopic breast cancer model 4T1. Tasquinimod treatment in this model reduced tumor growth and also the number of lung metastatic nodes after orthotopic injection of 4 T1 tumor cells into the mammary fat pad of Balb/c mice (Additional file 1: Figures S1A and S1B). As in the MC38-C215, the total population of tumor infiltrating CD11b-positive cells and the frequency of infiltrating CD11b^+^F4/80^+^ did not change after treatment, whereas a tasquinimod-induced shift from a CD206-positive to CD206-negative phenotype could be determined (Additional file 1: Figures S1C-E) and the shift sustained over time and seemed to be independent of tumor size (Additional file 1: Figures S1G and Fig. 1a). In contrast to the MC38-C215 tumors though, the infiltrating CD11b^+^ component in 4T1 tumors was mainly Gr1^+^ cells and Ly6C^low^Ly6G^+^ cells was the dominating population which was not affected by the tasquinimod treatment (Additional file 1: Figure S1F).

To study functional and genetic changes in the tumor-infiltrating macrophages we performed mRNA-analysis of the CD11b^+^F4/80^+^ cells by qRT-PCR. The CD11b^+^F4/80^+^ population from MC38-C215 tumors of treated mice had up-regulated mRNAs of genes characteristic of M1 macrophages (i.e. *Nos2, Cxcl9, Cxcl11, Il-12β* and *Il-6*) [33], whereas mRNA for genes typical for immunosuppressive cells of the M2 phenotype (*i.e. CD206 & Arg-1*; Fig. 2f and Additional file 7; Table S1) were down-regulated. Similarly, a decrease in the expression of different M2-associated genes (i.e CD206 & Arg-1; Additional file 1: Figure S1G) was observed in 4T1 tumors treated with tasquinimod for 14 or 28 days.

M2 TAMs have a high capacity to suppress T cell proliferation by several mechanisms, including high expression of arginase-1 (Arg-1) which acts to deplete L-arginine in the tumor. The funtionality of the CD11b^+^F4/80^+^ cells was investigated by determining their capacity to suppress T cell proliferation *ex vivo*. Naïve CD4^+^ T cells were stimulated with anti-CD3/CD28 antibodies in the presence of FACS-sorted CD11b^+^F4/80^+^ cells from treated or untreated MC38-C215 tumors. The analysis showed that macrophages from tasquinimod-treated mice were less able to suppress T cell proliferation compared to macrophages from control mice (Fig. 2g). These results combined show that the TAM population in tumors from tasquinimod-treated mice are functionally skewed towards the M1-like phenotype while control tumors contain a myeloid cell population dominated by M2 macrophages.

The number of splenic CD11b^+^ myeloid cells was significantly increased in MC38-C215 tumor bearing mice compared to non-tumor bearing mice, but in contrast to what was observed in the tumor, this expansion was prevented by tasquinimod treatment (Additional file 2: Figure S2A). However, in tasquinimod treated naïve mice the CD11b^+^ cell population did not significantly change in the spleen confirming that the observed increase in tumor bearing mice was a tumor related event inhibited by tasquinimod treatment (Additional file 2: Figure S2B).

### Changes in the tumor microenvironment are established within a week after initiating tasquinimod treatment

**Fig. 3:**
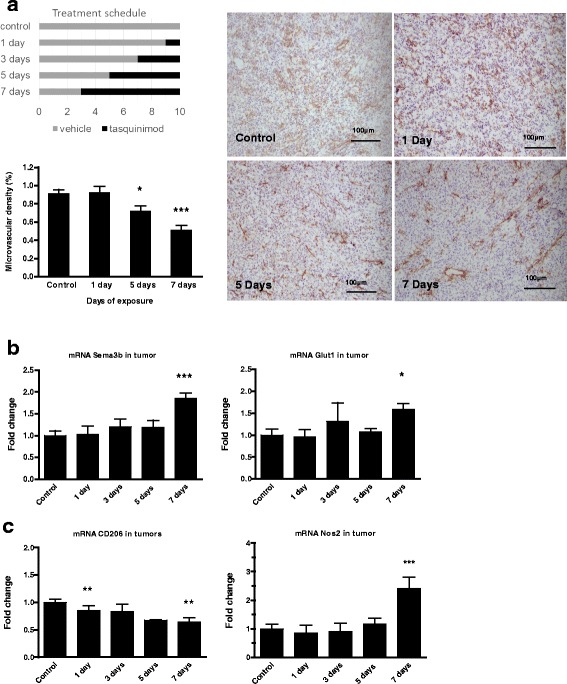
Tasquinimod induces tumor immunomodulation and inhibits tumor angiogenesis within one week of exposure. **a** CD31 staining of tumors from control and tasquinimod treated animals. Mice were treated for 1, 5 or 7 days with tasquinimod (p.o.; 30 mg/kg/day). **b** Hypoxia-induced expression of Hif-1α controlled genes in tumor samples; *SemaphorinB* (left panel) and *Glut-1* (right panel) after 7 days of exposure. **c** Time study of mRNA expression in tumors treated with tasquinimod for 1 up to 7 days of exposure *CD206* (*Mrc1*) (left panel) and *Nos2* (right panel) (versus control; * *p* < 0.05; ** *p* < 0.01 and *** *p* < 0.001; One way ANOVA with Dunnetts multiple comparison test, Error bars indicate s.e.m)

In order to map effects of tasquinimod on the TME, microvascular density was determined by anti-CD31 staining after treatment of established tumors for 1, 3, 5 or 7 days before termination of the experiment 10 days after inoculation (Fig. 3a, left upper panel). No significant anti-tumor effects were observed after short-term exposure (data not shown), whereas a significant reduction in vascular density was induced within 7 days of tasquinimod exposure (Fig. 3a). This was further emphasized by mRNA analysis of whole tumor preparations, where a significant elevation in hypoxia-related genes (i.e. *Glut-1 (Slc2a1), Angpt2, Stc2,* and *Semaphorin B*) could be seen coinciding with reduced CD31-staining (Fig. 3a and b; Additional file 8: Table S2). This indicates onset of an angiogenic switch concomitant with reduced microvascular density, increased hypoxia and induction of Hif-1α-controlled genes. Expression of *CD206* and *Nos2* were analyzed in tumor samples as markers of M2 and M1 related genes respectively. A decrease in CD206 (*Mrc1*) and an increase in *Nos2* (Fig. 3c; Additional file 8: Table S2) expression could be seen in the TME before or in connection with the changes in CD31 staining and hypoxia-regulated genes. Hence, tasquinimod-induced changes in the TME including vascularization, hypoxia and immune-related genes are established within the first week of treatment.

### Tasquinimod treatment results in phenotypic changes of myeloid cells in the tumor at early time points after exposure

**Fig. 4:**
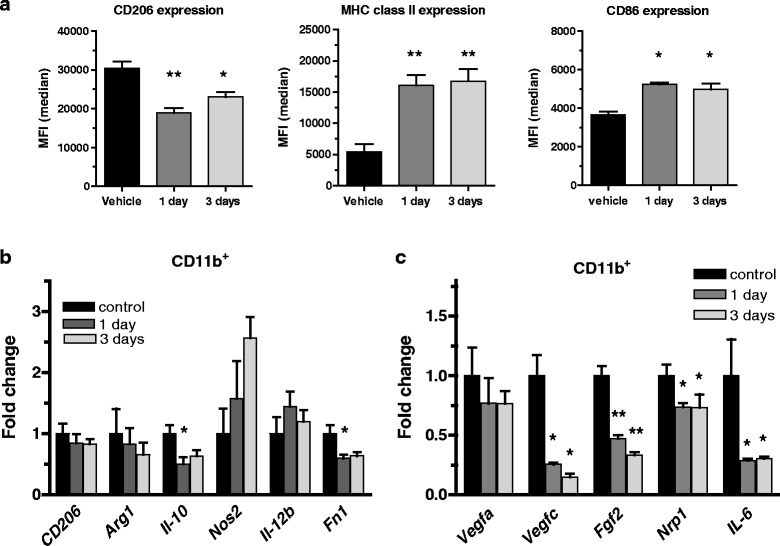
M1 macrophages, induced by tasquinimod modulates the expression of different angiogenic factors. **a** Median Fluorescence Intensity (MFI)values of CD206, MHC-II and CD86 cell surface expression evaluated by flow cytometry on isolated tumor infiltrating CD11b^+^ cells gated on the F4/80^high^ population after 1 and 3 days of in vivo exposure to tasquinimod. Results from one out of three representative experiments are shown. **b** Up-regulation of M1 genes *Nos2* (*iNos*) and *Il-12β*, and down-regulation of M2 genes *CD206*, *Arg-1* and *Il-10* mRNA expression in isolated tumor infiltrating CD11b^+^ cells after 1 and 3 days of in vivo exposure to tasquinimod. **c** Changes of pro-angiogenic genes *Vegfa, Vegfc, Fgf2*, *Nrp1 and Il-6* mRNA expression in isolated tumor infiltrating CD11b^+^ cells after 1 and 3 days of *in vivo* exposure to tasquinimod (versus control; * *p* < 0.05; ** *p* < 0.01 and *** *p* < 0.001; One way ANOVA with Dunnetts multiple comparison test, Error bars indicate s.e.m)

In order to study early changes in the myeloid cells specifically, analysis of purified CD11b^+^ cells from tumors treated with tasquinimod for 1 and 3 days was performed. Already after short-term exposure, the F4/80^+^ macrophages displayed reduced cell surface expression of CD206, and an upregulation of MHC class II and CD86 (Fig. 4a; Additional file 3: Figure S3), indicating an impact on the cellular activation state already after 1 day of exposure in vivo. The cellular changes were further emphasized when analyzing mRNA levels from the isolated cells. Here, a change in *Fn1* and *Il-10* expression was observed after 1 day of treatment while only minor changes in mRNA levels for *CD206* and *Arg-1* (Fig. 4b; Additional file 9: Table S3) could be seen. Interestingly, in the CD11b^+^ cells a significantly decreased expression of the pro-angiogenic factors *Vegfc, Fgf2, Nrp1* and *Il-6* was clearly evident at these early time points (Fig. 4c). These results show that tasquinimod treatment induces early changes of the myeloid cell population that lead to upregulation of inflammatory markers and a down-regulation of pro-angiogenic factors. These changes are consistent with an initiation of a change in phenotype from an M2-like TAM to a M1-like macrophage.

**Fig. 5:**
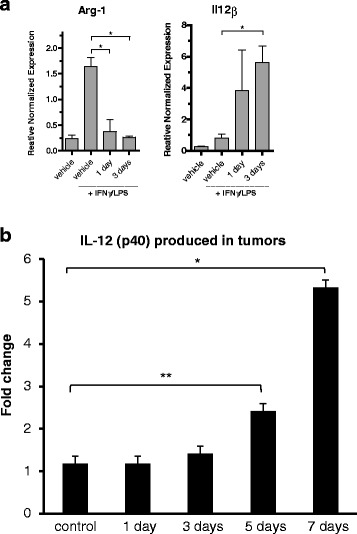
Tasquinimod-induced functional changes in tumor derived CD11b^+^. **a** Changes of *Arg-1* and *Il-12β* mRNA levels in isolated tumor infiltrating CD11b^+^ cells stimulated with LPS/IFNγ and cultured *ex vivo* for 24 h. Results from one out of three representative experiments are shown (* *p* < 0.05; *t*-test, Error bars indicate s.e.m). **b** IL-12(p40) cytokine production of tumor lysates prepared from treated and non-treated tumors (*p < 0.05 Jonckheere-Terpstra test; Error bars indicate s.e.m)

To further analyze the functional changes of the infiltrating CD11b^+^ cells after treatment, isolated cells from tumors were activated *ex vivo* by culturing for 24 h in medium ± LPS/IFNγ and induction of *Arg-1* or *Il12β* gene expression was studied. M1 macrophages produce IL-12 in response to this stimulation [33, 34] while arginase-1 is an effector molecule and signature of M2 macrophages. qRT-PCR analysis of cells after induction revealed that isolated CD11b^+^ cells from vehicle-treated MC38-C215 tumors expressed high levels of *Arg-1* mRNA while isolated cells from tasquinimod treated mice had completely lost the capability to induce *Arg-1* (Fig. 5a). On the other hand, CD11b^+^ cells from tasquinimod-treated mice induced high levels of *Il12β* whereas CD11b^+^ cells from vehicle did not respond at all to the same extent. This experiment shows that tasquinimod treatment induces functional changes of tumor associated myeloid cells already after 1-3 days of treatment (Fig. 5a).

In order to verify the relevance of these changes in vivo, protein determination by a multiplex assay (Millipore) was performed on tumor tissue lysates. 12 proteins were analyzed (Additional file 10: Table S4) and of these IL-12(p40) levels were significantly higher after 5 and 7 days of tasquinimod treatment (Fig. 5b). This was also accompanied by significantly increased serum levels of IL-12(p40) (Additional file 4: Figure S4).

## Discussion

Tumors devise different mechanisms to shape their microenvironment to allow growth and to evade anti-tumor immune responses. Among them, infiltrating TAMs promote tumor progression and metastasis [7] and are commonly recognized as obstacles to various anti-cancer therapies [35]. We recently showed that tasquinimod reduces the ability of suppressive myeloid cells, including TAMs, to support tumor growth and significantly enhances the efficacy of cancer immunotherapies [21]. To better understand the sequence of events regulated by tasquinimod to exert this immunomodulatory activity, we took advantage of the MC38-C215 colon cancer model in which F4/80^+^ macrophages constitute the dominant myeloid cell population [32]. Circulating monocytes are recruited by tumors, and depending on the inflammatory state of the tumors, they may differentiate into M1 or M2 macrophages, gaining specialized functional properties. Moreover, during tumor progression, MDSCs accumulate in blood and in spleen and are also recruited to the TME, where they accumulate and may also differentiate to M1 or M2 macrophages [36–38]. M1 macrophages produce higher levels of pro-inflammatory IL-12 and lower levels of anti-inflammatory IL-10 [39]. They also produce effector molecules, such as iNOS, which contribute to the Th1 response in the TME. Our findings show that tasquinimod changes the polarization of macrophages in the tumor by several different parameters, such as an increase in MHC class II, CD86 and *Nos2* expression and a reduced *arginase-1* and CD206 expression. These results confirm that tasquinimod induces a shift from immunosuppressive M2 macrophages to immunostimulatory M1 macrophages in the MC38-C215 tumor model, in agreement with previously published data in experimental models of prostate cancer and melanoma [21].

The increased expression of M1 markers in macrophages after tasquinimod treatment raises the question whether tasquinimod increases the recruitment of circulating monocytes and myeloid cells, and/or their differentiation into M1 macrophages and accumulation in the TME. Tumor infiltration of TAMs is often mastered by the Ccl2 chemokine that attracts Ccr2^+^ monocytes circulating in the blood [10], but in this study the expression of *Ccl2* mRNA (Additional file 8: Table S2) and its encoded protein MCP-1 (Additional file 10: Table S4) in tumors were not modulated by treatment and hence cannot explain the effects on TAMs. On the other hand, S100A9 is also known to affect migration of myeloid cells and MDSCs in particular into tumor tissue [29]. Thus binding of tasquinimod to S100A9, and thereby inhibiting its interaction with receptors on myeloid cells, is a possible mechanism for an altered migration of myeloid cells into the tumor. In the MC38-C215 tumors, however, the myeloid compartment of the TME was dominated by F4/80^+^ macrophages and this composition was stable over time and with respect to tumor size (from day 7 to day 14, data not shown). Since the effect of tasquinimod treatment was a change from F480^+^CD206^+^ to F4/80^+^CD206^−^ macrophages without changing the total frequency of F4/80^+^ cells, this would require a rapid turnover of macrophages in the tumor to explain the treatment effects. However, since M2 macrophages are known to be a relatively stable subset of myeloid cells in the TME not undergoing constant replenishment [7, 40] we strongly believe this is unlikely and that tasquinimod rather induces a rapid change in polarization of the existing macrophages in the tumor. Instead, S100A9 is known to induce NF-κB activity [41], and targeting NF-κB in colon cancer cells educates macrophages toward a M1-like phenotype and inhibits peritoneal metastasis [42]. These latter data support a hypothesis where S100A9 sustains NF-κB activation by interaction with receptors such as TLR4, and where tasquinimod by blocking S100A9-receptor interactions, decreases NF-κB activity and induces a polarization of macrophages toward the M1 phenotype. Moreover, rapid changes from a M2 to a M1 phenotype of infiltrating macrophages was reported by Guiducci et al. [43], that in Ccl16-treated TSA mammary carcinoma tumors observed changes in polarization within less than 16 h, when challenging TLR9 signaling by treatment with CpG and an anti-IL-10 receptor antibody. Taken together our findings suggest that tasquinimod may not primarily trigger a difference in the recruitment of F4/80^+^ myeloid cells, but may rather change their differentiation state within the MC38-C215 tumors. Irrespective of the diversity of myeloid cells present in the tumor microenvironment, we demonstrated that tasquinimod was able to induce a long-term education of macrophages from a M2 to a M1 phenotype in 4T1 tumors. These findings were associated with a prolonged inhibition of 4T1 tumor growth over 28 days and with a reduction in lung metastasis development.

Although reduced immunosuppression is one consequence of macrophage M1-polarization, tasquinimod inhibited MC38-C215 tumor growth in immune deficient nude mice by the same magnitude as in the wild type mice (Additional file 5: Figure S5), indicating that the tasquinimod-induced antitumor effect is not due to a change in the T cell response. In accordance with this, whilst tasquinimod did affect the suppressive potential of MC38-C215 TAMs this did not lead to an increased recruitment of T cells into the tumors *in vivo*. However, a number of factors influence T-cell tumor infiltration and TAMs may not be the prime obstacle for T-cells to reach the MC38-C215 tumors. Indeed, several studies from the literature have reported that the expression of immune checkpoints inihbitors in the TME such as the PD-1/PD-L1 axis may limit T cell infiltration and expansion [44].

Instead, substantial changes in vascularization in the tumor could be observed after a week of treatment with tasquinimod. Since CD11b^+^MHC-II^low^ cells previously have been shown to be pro-angiogenic in different mouse tumor models with a common gene profile [33], the shift from a MHC-II^low^ into a MHC-II^high^ profile seen in this study also reflects a change into TAMs with a less pro-angiogenic M1 phenotype after treatment with tasquinimod (Additional file 9: Table S3). Interestingly, 24 h after tasquinimod exposure, mRNA expression of different angiogenic factors (*Vegfc, Nrp-1, Fgf2, Il-6*) was decreased in MCH-II^high^ macrophages compared to vehicle TAMs, suggesting that M1 macrophages contribute to tumor vasculature shrinkage. Indeed, using syngeneic mice bearing MC38-CEA tumors, Farsaci et al. showed the presence of CD31^+^ and CD105^+^ tumor vessels formed after transferring CD11b^+^ myeloid cells, confirming that tumor vessels are modulated by cells of monocytic origin [32]. Furthermore, it has recently been shown that tumor-derived S100A9 induced the production of IL-6 by myeloid cells in ovarian tumors [45], and that IL-6 drives angiogenesis with defective pericyte coverage typical for TME [46]. Moreover, different cellular assays showed that tasquinimod did not inhibit the kinase driven angiogenesis from the following proteins: EGF-R, FGF-R2, IGF1-R, c-kit, Met, SRC, Tie2, VEGF-R2 and VEGF-R3 (Additional file 11: Table S5). Also, tasquinimod was not able to suppress VEGF-A induced angiogenesis *in vitro*, in contrast to anti-VEGF or suramin treatment (Additional file 6: Figure S6). These data validate that tasquinimod has a unique mechanism of action that is completely different from classical anti-angiogenic and tyrosine kinase inhibitor drugs. In addition to S100A9 and its importance for macrophage signaling and angiogenesis, an alternative molecular target for tasquinimod is HDAC4, where the inhibition of HDAC4 interaction with NCoR/HDAC3 has been shown to inhibit the transcription of HIF-1α-controlled genes such as *VegfA* [31]. HIF-1α signaling in response to hypoxia has been described as important for myeloid cell function in the TME, but although tumor hypoxia and HIF-1α regulate MDSC differentiation [10, 47], hypoxia does not seem to be the major driver of TAM differentiation into pro-angiogenic M2 subsets. Instead, HIF-1α specifically regulates expression of pro-angiogenic factors such as VegfA and Glut1 in already differentiated M2 TAMs [37, 48]. Thus, defective microvessel formation in treated MC38-C215 tumors are likely related to down-regulated expression of a set of pro-angiogenic genes in macrophages. This report is the first to unveil the sequence of events regulated by tasquinimod demonstrating that immunomodulatory properties of the tumors are the first to be modulated after exposure, which in turn may drive its anti-angiogenic activity.

To gain more insight into macrophage driven angiogenesis inhibition in our experimental model, macrophages derived from tumors exposed to tasquinimod for 1 day or 3 days were stimulated with the classical M1 activators LPS and IFNγ. Interestingly, *Arginase-1* mRNA expression was substantially decreased after tasquinimod treatment, indicating a shift of TAMs into the M1 phenotype. Li et al. showed that over-expression of Arginase-1 increased HUVEC proliferation and promoted tube-like morphogenesis, suggesting that Arginase-1 plays an important role in the process of angiogenesis [49]. Moreover, an increase in *Il-12β* expression was observed in macrophages treated with tasquinimod. The mechanisms of Il-12-mediated antitumor activity depend not only on activation of innate and adaptive effector immune mechanisms, but also on inhibition of angiogenesis [14, 15, 51]. In tumors treated with tasquinimod, production of IL-12 (p40) was high in the TME after 5 to 7 days of tasquinimod exposure with a concomitant decrease in CD31 immunostaining.

Macrophages exert their antitumor effects by i) producing anti-angiogenic and cytotoxic cytokines and by ii) orienting the adaptive immune response by presenting MHC-II–bound peptides to T cells [11, 51]. Tasquinimod modulates both mechanisms triggered by macrophages to mediate antitumor activity. Thus, tasquinimod treatment impairs tumor growth and is associated with a reduction in the microvascular density of MC38-C215 tumors. An early switch of macrophages may precede several mechanisms that are involved in tumor progression and metastasis. We demonstrated here that the anti-angiogenic as well as the anti-metastatic effects of tasquinimod are preceded by an early switch of tumor macrophages into a less suppressive and anti-angiogenic population. This was manifested by a decrease of pro-angiogenic markers and the increase of anti-angiogenic IL-12 production in MC38-C215 tumors. However, further analysis are needed to address the direct link between the immunosuppressive properties of myeloid cells and their ability to inhibit tumor vessel formation and metastatic spread.

## Conclusions

In this study, we demonstrate a significant antitumor effect by tasquinimod treatment which associates with early effects on tumor infiltrating myeloid cells leading to a change in phenotype from pro-angiogenic and immunosuppressive M2-like TAMs to pro-inflammatory M1-like macrophages. These changes are consistent with the effects of tasquinimod previously seen on tumor vascularization, immune suppression and metastasis [21, 24] and highlights tasquinimod as an immunomodulatory anti-tumor agent. Modulating tumor-infiltrating macrophages and other myeloid cells is an important piece in establishing therapeutic strategies to overcome tumor immune evasion, progression and metastasis development. Our findings further highlight the potential of educating macrophages to eradicate tumors and the potential of potent modulation of myeloid cells in the TME.

## Methods

### Animals and cells

Female C57Bl/6 mice were purchased from Taconic Europe A/S (Denmark), and maintained under standardized conditions. Female Balb/c mice were purchased from JANVIER LABS (France). The mice were routinely used at the age of 8 to 12 weeks. All studies were approved by the Bioethics Committee in Lund, Sweden (M60-10 and M450-12), the Bioethics Committee of IPSEN (C2EA-13-048-S1) and by the French ministry (00617.01). All the experiments were conducted in accordance with the NIH Guide for the Care and Use of Laboratory Animals. Mice were treated with tasquinimod (30 mg/kg) *ad lib. via* drinking water or in the case of time studies by *p.o.* administration. The 4 T1 tumor cells were purchased from ATCC (France). The MC38 tumor cell line was obtained from S. A. Rosenberg (Nat. Cancer Inst., Bethesda, Maryland) and was transfected with the C215 antigen as previously described [52]. The cells were cultured in RPMI-1640 supplemented with ultra-glutamine (BioWhittaker/Lonza, Wokingham, UK); 10 % fetal bovine serum (Fisher Scientific, Pittsburgh, PA), 1 mM sodium pyruvate, 10 mM HEPES, 0.2 mg/ml gentamicine sulfate and 50 μM β-mercaptoethanol (R10 medium).

### In vivo tumor experiments

MC38-C215 cells were cultured as described above, counted and re-suspended and maintained in ice cold matrigel (BD Biosciences, San Jose, CA) at a concentration of 5 × 10^6^ cells/ml for subcutaneous inoculation. The tumor cells were implanted s.c. into the hind flank of mice on day 0 in a volume of 0.1 ml matrigel. 4T1 cells (10^5^) were injected orthotopically into the fourth mammary fat pad of Balb/c mice. Mice were treated *ad lib* or in the case of time studies by *p.o.*with tasquinimod dissolved in the drinking water (30 mg/kg) for indicated times. The tumor size was measured by a caliper twice a week. Tumor volume (V) was calculated using the following equation: (width)^2^ × length/2, both in millimeters.

### Cell sorting and FACS analysis

Single cell suspensions were prepared from spleens and from tumors by incubating the tumors cut into small pieces in 1.6 mg/ml Collagenase IV (Worthington Biochemical Corporation, Lakewood, NJ) and 0.1 % DNase (Sigma-Aldrich, St. Louis, MO) for 45 min at 37 °C, followed by meshing the tumors in a 70 μm cell strainer. CD11b^+^ cells were further purified from tumor cell suspensions by magnetic depletion of MC38-C215 tumor cells after incubation with anti-C215-biotin (10 μg/ml) and Dynabeads® Biotin Binder beads (Life Technologies) followed by positive selection of CD11b^+^ cells by MACS technology (Miltenyi).

Before incubating the cells with specific fluorochrome-labeled antibodies, Fc-receptors were blocked using anti-CD16/CD32 mAb (clone 2.4G2; BD Biosciences). The following fluorochrome-labeled antibodies were used: CD11b (clone M1/70), Ly6G (clone 1A8), Ly6C (clone AL-21), F4/80 (clone BM8), CD206 (clone C068C2), CD86 (clone GL1), MHC II (I-A/I-E; clone M5/114.15.2), CD4 (clone RM4-5), and CD8a (clone 53-6.7), purchased from BD Biosciences (San Jose, CA), eBioscience (San Diego, CA) and BioLegend (San Diego, CA). Intracellular staining of CD206 was performed using the Mouse Regulatory T cell Staining Kit from eBioscience. Flow cytometric analysis was performed according to standard settings on a FACS CantoII flow cytometer (BD Biosciences).

### Histology

Tumors were sampled at different time points (1, 3, 5, or 7 days after initiation of treatment), weighted, embedded in OCT (Optimal Cutting Temperature) compound and snap frozen in pre-cooled isopentane in liquid nitrogen before storage at -80 °C. Tumors were sectioned for protein extraction (one 1000 μm-thick section), RNA extraction (one 100 μm thick section) or histology (8 μm thick sections) using a cryostat (Leica, CM 3050S). One slide per tumor was stained with HE in order to evaluate tumor morphology. CD31 immunohistochemical staining was performed with rat anti-CD31 monoclonal antibody (AbD Serotec, MCA1212) and peroxidase/diamminobenzidine revelation. Vascular density was evaluated for 3 regions of interest per tumor by measuring the percentage of stained area by image analysis using Image J software (MBF Image J 1.34 m). Formalin-fixed, paraffin-embedded mouse lung tissue sections were prepared and 5 μm sections were examined using H&E staining. Slides were scanned (Excilone - France) and image analysis was performed using a software, Halo (Indica Labs). Tumor infiltration in the lungs was characterized by manual segmentation of tumor nodules on digital whole slide at *×*2 magnification. Metastatic burden was quantified by counting the total number of metastatic lung nodes in each section.

### *In vitro* proliferation assays

#### Anti-proliferation effects *in vitro*

MC38-C215 cancer cells (5000 cells/well) were seeded in 96 well plates and the cultured cells treated with different concentrations (1–10 μM) of tasquinimod for 72 h under either normoxic or hypoxic growth conditions (1 % O_2_ in a hypoxia incubator chamber; STEMCELL Technologies, Grenoble, France). Viability was determined by MTT assay at the end of incubation.

#### *Ex vivo* suppression assay

CD4^+^ cell isolation: Naïve mouse spleens were pooled and after blocking with Fc-block, incubated for 20 min with 3 ml MACS buffer + 300 μl CD4-microbeads. The cells were washed with MACS buffer and resuspended in 2 ml MACS buffer prior to addition to an LS column (LS+ for MidiMACS; Miltenyi). The column was washed with MACS buffer and the CD4^+^ cells were eluted. The cells were then added to 96-well plates for suppression assay cultures. Suppression assay: CD4^+^ T cells isolated from naïve mice (*n* = 3) were stimulated with Dynabeads Mouse T-activators anti-CD3/-CD28 in the presence of infiltrating F4/80^+^ cells, sorted by FACS from treated and untreated MC38 tumors, in ratios of 1:1, 1:2, 1:4 and 1:8 (F4/80:CD4). Cell proliferation was measured by [^3^H]-thymidine incorporation. Pulsing was performed for 18 h with 0.5 μCi [^3^H]-dThd after 48 h incubation.

### Quantitative Real-time PCR (qRT-PCR)

RNA was extracted from FACS-sorted F4/80^+^ cells that were pre-isolated with anti-CD11b^+^ magnetic beads fractions of single cell suspensions of tasquinimod treated and untreated MC38-C215 tumors. RNA extraction of CD11b^+^ was performed using the RNeasy micro or mini kit according to the cell number (Qiagen, Hilden, Germany). RNA in tumors was isolated from 100 μm of OCT-embedded tumor cryosections using Trizol Reagent (Life Technologies). RNA concentration and purity was determined through measurement of A260/A280 ratios with a NanoDrop ND-1000 spectrophotometer. RNA integrity was checked using the Agilent 2100 Bioanalyzer. cDNA was prepared using the iScript kit (BioRad, Hercules, CA, USA), or High-Capacity cDNA Reverse Transcription Kit (Life Technologies) following the manufacturer’s instructions. QPCR was performed with a two step PCR-protocol (95 °C for 10 min, followed by 45 cycles of 95 °C for 10 s and 58 °C for 30 s) using SYBR Green (SsoFast EvaGreen; BioRad) or 95 °C for 10 min, followed by 40 cycles of 95 °C for 10 s and 60 °C for 1 min using Taqman gene expression (Life Technologies). Expression levels were calculated as normalized ΔCt expression values between target gene and the two “housekeeping” genes *β-Actin* and *Yhawz* for Sybergreen technology (Additional file 7: Table S1), and *Hmbs* and *Cyclophilin A* for Taqman technology (Additional file 9: Table S3). The primer sequences used for target genes, and the Taqman probes references are listed in Additional files 8, 9 and 12: Tables S2, S3 and S6.

Data were presented as fold induction (2ΔΔCt) of treated tumors compared to control tumors levels (ΔΔCt).

#### Gene expression ex vivo

Purified CD11b^+^ cells was cultured *ex vivo* for 24 h in DMEM medium ± IFNγ (2 ng/ml) and LPS (100 ng/ml) in order to induce M1 macrophages to express *Il-12β* or M2 macrophages to express *Arg-1* [33, 34]*.*

### Cytokine determination by multiplex assay

Cytokines were extracted from 1 mm thick section of frozen-embedded tumors, After thawing and 3 washes in PBS, the pellet is suspended in PBS + Protease Inhibitor Cocktail (Roche) and ground by ceramic beads in a homogenizer (Fastprep, MP bio). Homogenate was then passed through Qiashredder column (Qiagen) to reduce viscosity. The different samples were assayed for protein concentration and adjusted at 2 mg of protein/ml.

Cytokines were measured directly in nondiluted or 1/20 diluted tumor homogenate or in nondiluted mice serum using Multiplex Immuno-assay kits (Milliplex kit, Merck-Millipore) following manufacturer instructions. Signal detection was performed on Luminex 200 (Luminex) and MFI (Median Fluorescence Intensity) was recorded. The quantification for each cytokine was detemined using standard curve analyzed with a 5 Parameter Logistic model (XLfit software, version 5.3, IDBS). For each cytokine in each assay, Low Limit of Detection (LLOD) and Quantification (LLOQ) were calculated. Statistical analysis on cytokine levels were perfomed using Wilcoxon test or Jonckheere-Terpstra test when the lower values were between LLOD and LLOQ.

### Statistical analysis

Normal data distribution was evaluated using the Shapiro-Wilk test. The difference in tumor growth over time between the two treatment groups was statistically evaluated by 2-way ANOVA analysis. In *ex vivo* experiments, analysis of groups with different treatment was analyzed using One-way ANOVA with Dunnetts multiple comparison test or two-tailed student’s *t*-test. For non-normal distributed data the non-parametric Kruskal-Wallis or Mann-Whitney test was used. Statistical analysis on cytokine levels were perfomed using Wilcoxon test or Jonckheere-Terpstra test when the lower values were between LLOD and LLOQ. A *p* value less than 0.05 was considered statistically significant (* = *p* < 0.05; ** = *p* < 0.01; *** = *p* < 0.001).

## Additional files

Additional file 1: Figure S1. Tasquinimod inhibits (A) the growth of 4T1 tumors and (B) metastasis at the dose of 30 mg/kg for 28 days of treatment. The number of lung metastases per paraffin section (****p* < 0.001; *t*-test). The modulation of different subsets of infiltrating CD11b^+^ cells within the tumor microenvironement was monitored by FACS analysis after 28 days following tumor cell inoculation. (C) Percent CD11b^+^ cells of infiltrating cells and (D) F4/80^+^ cells as frequency of total CD11b^+^ cells. (E) Median Fluorescence Intensity (MFI) of CD206^+^ gated on CD11b^+^ F4/80^+^, and (F) frequency of GR1^+^ cells of total CD11b^+^ cells; Ly6C^low^Ly6G^−^, Ly6C^high^Ly6G^−^ and Ly6C^low^Ly6G^+^ as frequency of total CD11b^+^ cells. (G) A sustained shift in CD206 population over time (**p* < 0.05; two-way ANOVA, Error bars indicate s.e.m). (H) Down-regulation of M2 markers in 4T1 tumors after tasquinimod treatment at days 14 and 28, (versus control; **p* < 0.05, ***p* < 0.005, ****p* < 0.001; two-way ANOVA, Error bars indicate s.e.m). (PDF 71 kb) Additional file 2: Figure S2. Modulation of myeloid CD11b^+^ subpopulations in spleen by tasquinimod. (A) Total CD11b^+^ cells in the spleen from tumor bearing mice treated or non-treated with tasquinimod in comparison to naïve non-treated mice, and (B) total CD11b^+^ cells in the spleen from naïve mice after treatment with tasquinimod (**p* < 0.05 and ***p* < 0.01; *t*-test, Error bars indicate s.e.m). (PDF 15 kb) Additional file 3: Figure S3. Median Fluorescence Intensity (MFI) plots of CD206, MHC-II and CD86 cell surface expression evaluated by flow cytometry on isolated tumor infiltrating CD11b^+^ cells gated on the F4/80^high^ population after 1 and 3 days of in vivo exposure to tasquinimod. Representative plots from one experiment are shown and dotted line indicate aproximal median value for vehicle (**p* < 0.05 and ***p* < 0.01; One-way ANOVA with Kruskal-Wallis, Error bars indicate s.e.m). (PDF 79 kb) Additional file 4: Figure S4. Serum levels of the IL-12(p40) cytokine in tumor bearing mice exposed to tasquinimod for 1 up to 7 days (* *p* < 0.05; *t*-test, Error bars indicate s.e.m). (PDF 11 kb) Additional file 5: Figure S5. Tasquinimod inhibits MC38-C215 tumor growth in a T cell independent manner. Nude mice were inoculated s.c. with 0.5 × 10^6^ MC38-C215 cells. Treatment with tasquinimod (30 mg/kg ad lib.) was initiated on the day of tumor inoculation and continued throughout the experiment (**p* < 0.05; 2-way ANOVA). Tumor volume (left panel) measurements and tumor weight at the end of experiment (**p* < 0.05; Mann Whitney) (right panel). (PDF 11 kb) Additional file 6: Figure S6. Endothelial Colony Forming cells were cultured on a layer of Adipocytes Stem cells maintened overnight at 37 °C following Angiokit instructions (Essen Biosciences). Tasquinimod was added in each well at different concentrations ranging from 1.56 to 50 μM in the presence of 4 ng/ml of recombinant VEGF-A (R&D). The tube lengths of the formed vessels were monitored by Incucyte for at least 80 h (Essen BioScience). Suramin or Anti-VEGF (R&D) are used as positive controls for inhibition of tube vessel formation induced by VEGF-A. (*** *p* < 0.001; 2-way ANOVA, Error bars indicate s.e.m). (PDF 28 kb) Additional file 7: Table S1. Average raw Ct values for the different genes analyzed in F4/80^+^ population using Syber Green Technology (PDF 29 kb) Additional file 8: Table S2. Gene expression profiling in tumors treated with tasquinimod after 1, 3, 5 and 7 days of exposure using Taqman technology (Life technolgies). The results were presented as fold change of treated tumors to control group levels. (PDF 95 kb) Additional file 9: Table S3. Gene expression profiling in CD11b^+^ macrophages derived from tumors treated with tasquinimod after 1 and 3 days of exposure using Taqman technology (Life Technolgies). The results were presented as fold change of treated tumors to control group levels. (PDF 106 kb) Additional file 10: Table S4. Fold change in protein expression levels of treated tumors compared to control group after 1, 3, 5 and 7 days of exposure using Luminex Technology. (*p* < 0.05; One way ANOVA). (PDF 44 kb) Additional file 11: Table S5. (A) Summary of tested kinases in different cellular assays. The respective cell line, ligand, ligand concentration, and duration of stimulation with the ligand are indicated. (B) Cellular IC_50_ values for indicated compounds on the inhibition of EGF-R, FGF-R2, IGF1-R, KIT, MET, SRC, TIE 2, VEGF-R2 and VEGF-R3 kinase activity. Each compound was tested in 8 different concentrations ranging from 33.0 × 10^−11^ to 1.0 × 10^−5^ (in duplicate). (PDF 70 kb) Additional file 12: Table S6. List of probes used for mRNA quantification using Syber Green technology. (PDF 26 kb)

## Abbreviations

DAMP: damage associated molecular pattern
HDAC4: histone-deacetylase-4
HIF1α: hypoxia-inducible factor 1, alpha subunit
IKKβ: IκB kinase β
MDSCs: myeloid derived suppressive cells
NF-кB: nuclear factor kappa B
TAMs: tumor associated macrophages
TEM: tumor microenvironment
