# Supplementary figures and images for: Tasquinimod triggers an early change in the polarization of tumor associated macrophages in the tumor microenvironment
Source: J Immunother Cancer. 2015 Dec 15;3:53. doi: 10.1186/s40425-015-0098-5 (PMC4678646; doi:10.1186/s40425-015-0098-5)

Figure S1

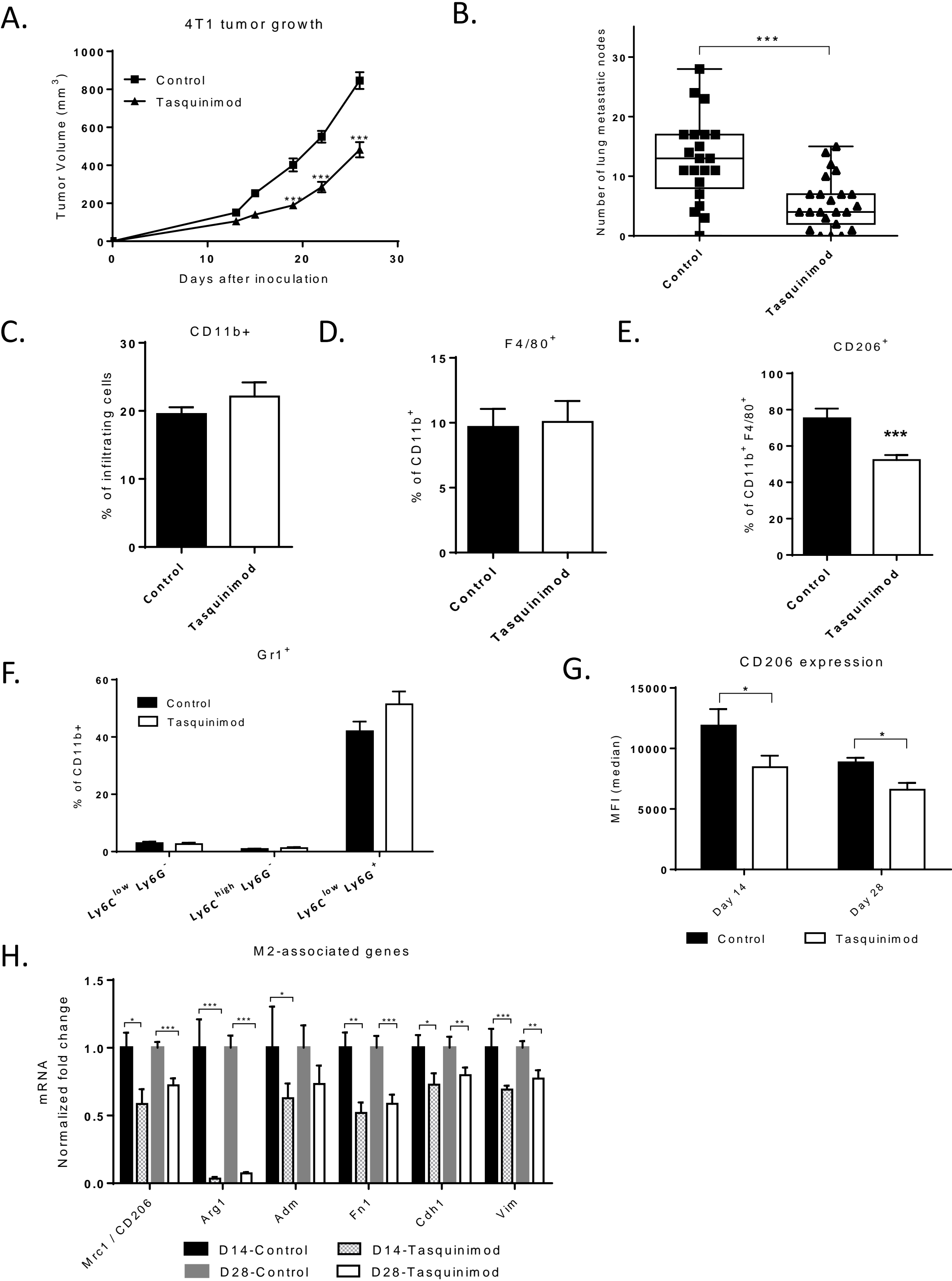

Supplement: Additional file 1: Figure S1. — Tasquinimod inhibits (A) the growth of 4T1 tumors and (B) metastasis at the dose of 30 mg/kg for 28 days of treatment. The number of lung metastases per paraffin section (***p < 0.001; t-test). The modulation of different subsets of infiltrating CD11b+ cells within the tumor microenvironement was monitored by FACS analysis after 28 days following tumor cell inoculation. (C) Percent CD11b+ cells of infiltrating cells and (D) F4/80+ cells as frequency of total CD11b+ cells. (E) Median Fluorescence Intensity (MFI) of CD206+ gated on CD11b+ F4/80+, and (F) frequency of GR1+ cells of total CD11b+ cells; Ly6ClowLy6G−, Ly6ChighLy6G− and Ly6ClowLy6G+ as frequency of total CD11b+ cells. (G) A sustained shift in CD206 population over time (*p < 0.05; two-way ANOVA, Error bars indicate s.e.m). (H) Down-regulation of M2 markers in 4T1 tumors after tasquinimod treatment at days 14 and 28, (versus control; *p < 0.05, **p < 0.005, ***p < 0.001; two-way ANOVA, Error bars indicate s.e.m). (PDF 71 kb) [file 40425_2015_98_MOESM1_ESM.pdf]

Figure S2

A.

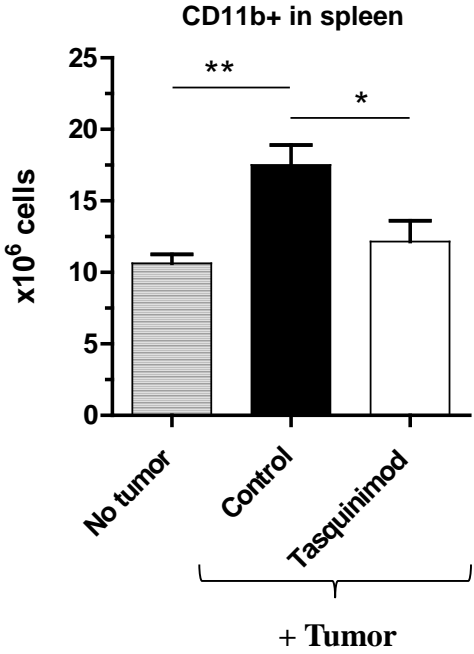

B.

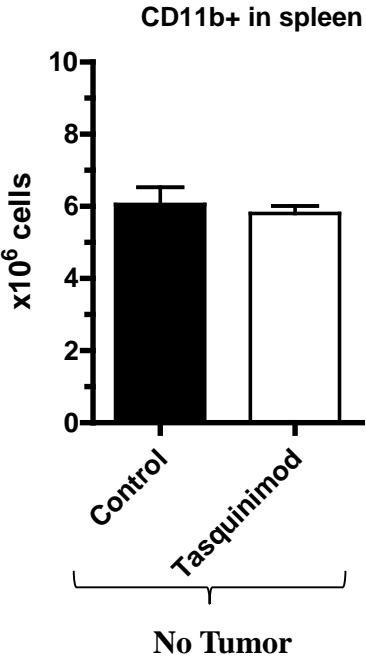

Supplement: Additional file 2: Figure S2. — Modulation of myeloid CD11b+ subpopulations in spleen by tasquinimod. (A) Total CD11b+ cells in the spleen from tumor bearing mice treated or non-treated with tasquinimod in comparison to naïve non-treated mice, and (B) total CD11b+ cells in the spleen from naïve mice after treatment with tasquinimod (*p < 0.05 and **p < 0.01; t-test, Error bars indicate s.e.m). (PDF 15 kb) [file 40425_2015_98_MOESM2_ESM.pdf]

Figure S3

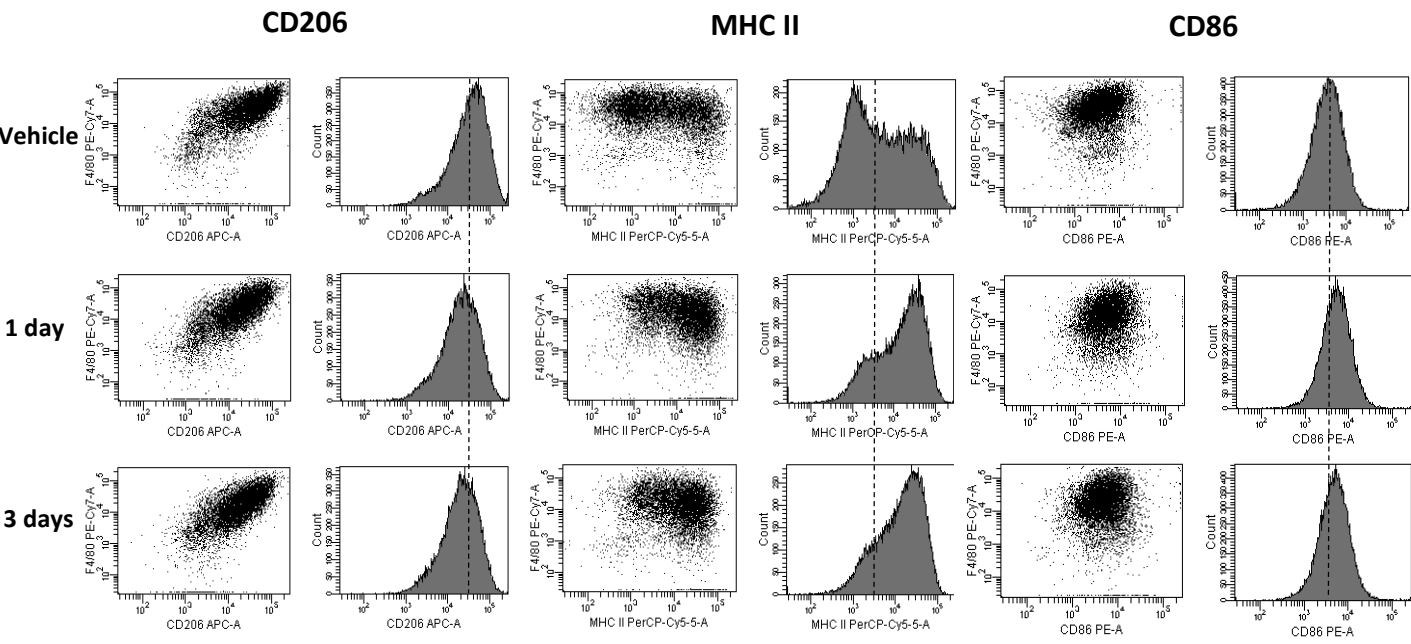

Supplement: Additional file 3: Figure S3. — Median Fluorescence Intensity (MFI) plots of CD206, MHC-II and CD86 cell surface expression evaluated by flow cytometry on isolated tumor infiltrating CD11b+ cells gated on the F4/80high population after 1 and 3 days of in vivo exposure to tasquinimod. Representative plots from one experiment are shown and dotted line indicate aproximal median value for vehicle (*p < 0.05 and **p < 0.01; One-way ANOVA with Kruskal-Wallis, Error bars indicate s.e.m). (PDF 79 kb) [file 40425_2015_98_MOESM3_ESM.pdf]

Figure S4

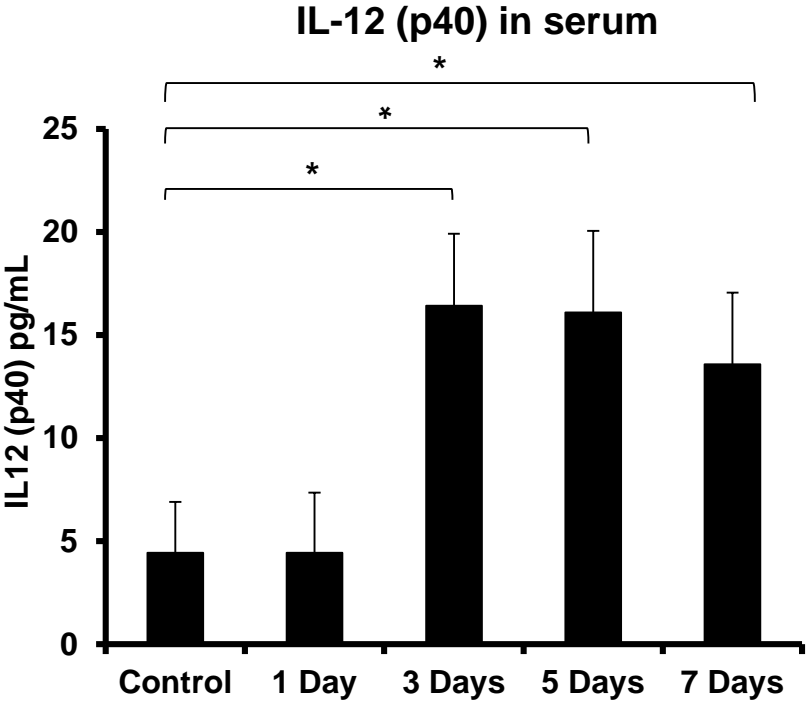

Supplement: Additional file 4: Figure S4. — Serum levels of the IL-12(p40) cytokine in tumor bearing mice exposed to tasquinimod for 1 up to 7 days (* p < 0.05; t-test, Error bars indicate s.e.m). (PDF 11 kb) [file 40425_2015_98_MOESM4_ESM.pdf]

Figure S5

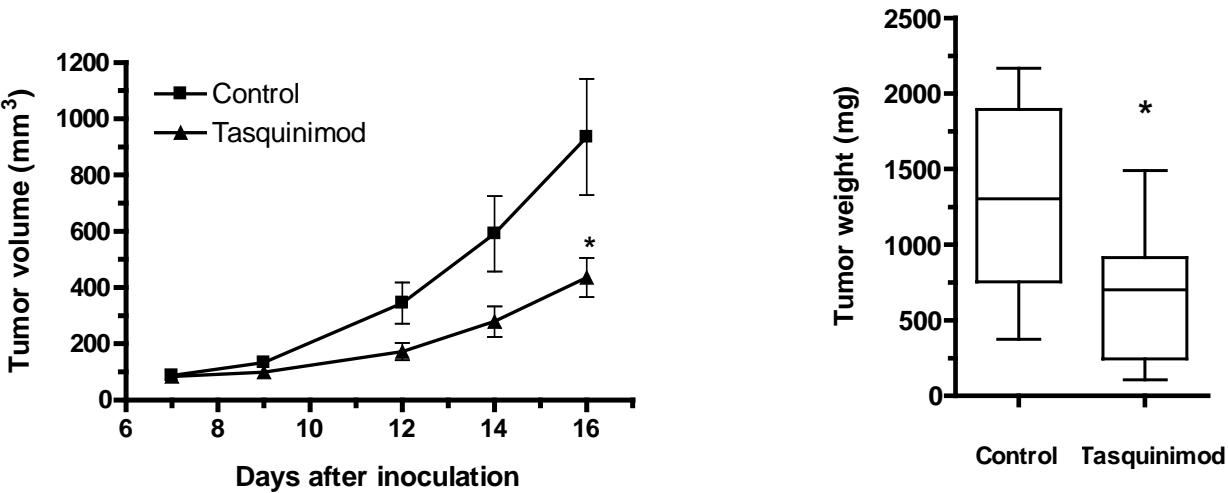

Supplement: Additional file 5: Figure S5. — Tasquinimod inhibits MC38-C215 tumor growth in a T cell independent manner. Nude mice were inoculated s.c. with 0.5 × 10^6^ MC38-C215 cells. Treatment with tasquinimod (30 mg/kg ad lib.) was initiated on the day of tumor inoculation and continued throughout the experiment (*p < 0.05; 2-way ANOVA). Tumor volume (left panel) measurements and tumor weight at the end of experiment (*p < 0.05; Mann Whitney) (right panel). (PDF 11 kb) [file 40425_2015_98_MOESM5_ESM.pdf]

## Figure S6

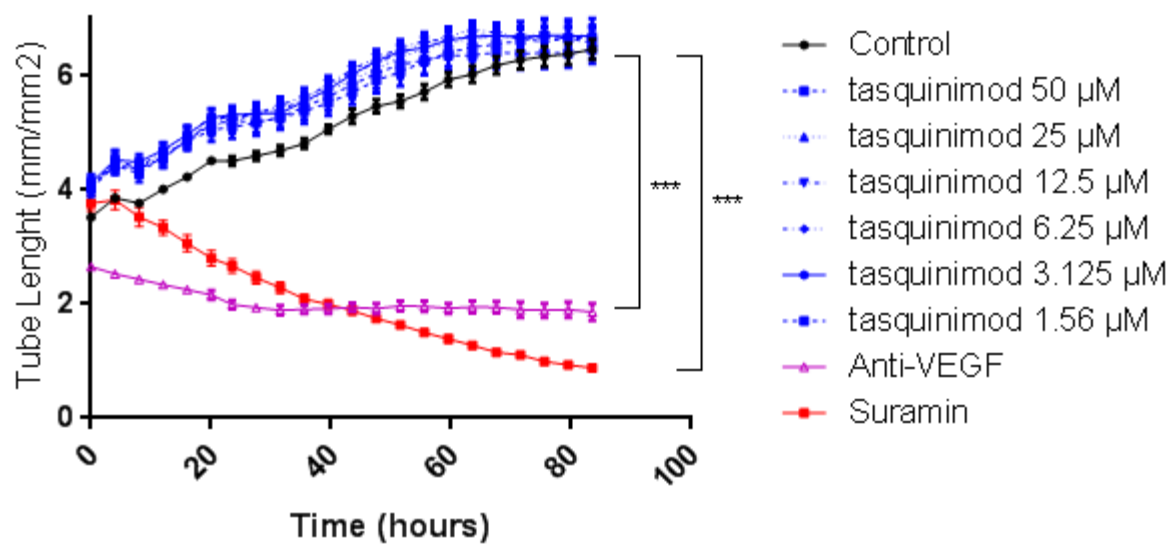

Supplement: Additional file 6: Figure S6. — Endothelial Colony Forming cells were cultured on a layer of Adipocytes Stem cells maintened overnight at 37 °C following Angiokit instructions (Essen Biosciences). Tasquinimod was added in each well at different concentrations ranging from 1.56 to 50 μM in the presence of 4 ng/ml of recombinant VEGF-A (R&D). The tube lengths of the formed vessels were monitored by Incucyte for at least 80 h (Essen BioScience). Suramin or Anti-VEGF (R&D) are used as positive controls for inhibition of tube vessel formation induced by VEGF-A. (*** p < 0.001; 2-way ANOVA, Error bars indicate s.e.m). (PDF 28 kb) [file 40425_2015_98_MOESM6_ESM.pdf]
