# Supplementary material for: Tasquinimod triggers an early change in the polarization of tumor associated macrophages in the tumor microenvironment
Source: J Immunother Cancer. 2015 Dec 15;3:53. doi: 10.1186/s40425-015-0098-5 (PMC4678646; doi:10.1186/s40425-015-0098-5)
Supplement: Additional file 7: Table S1. — Average raw Ct values for the different genes analyzed in F4/80+ population using Syber Green Technology (PDF 29 kb) [file 40425_2015_98_MOESM7_ESM.pdf]

Table S1

C_t values for genes analyzed in the F4/80$^+$ cell population

Gene	C_t control Average	SEM	C_t tasquinimod Average	SEM
Actin	21.13	0.07	22.00	0.07
Ywhaz	26.40	0.10	27.22	0.06
CD206 (Mrc1)	25.60	0.10	27.56	0.11
Arg-1	27.45	0.24	29.18	0.23
iNos (Nos2)	35.22	0.16	32.78	0.14
Cxcl9	37.75	0.23	37.95	0.11
Cxcl11	36.43	0.25	35.38	0.19
Il-12β	32.77	0.23	32.72	0.13
Il-6	37.54	0.15	37.54	0.16
