# Supplementary material for: Tasquinimod triggers an early change in the polarization of tumor associated macrophages in the tumor microenvironment
Source: J Immunother Cancer. 2015 Dec 15;3:53. doi: 10.1186/s40425-015-0098-5 (PMC4678646; doi:10.1186/s40425-015-0098-5)
Supplement: Additional file 8: Table S2. — Gene expression profiling in tumors treated with tasquinimod after 1, 3, 5 and 7 days of exposure using Taqman technology (Life technolgies). The results were presented as fold change of treated tumors to control group levels. (PDF 95 kb) [file 40425_2015_98_MOESM8_ESM.pdf]

Table S2

Fold changes in gene expression in tumors after 1, 3, 5 and 7 days of exposure

		Day 1		Day 3		Day 5		Day 7	
Mouse Gene	Assay Reference	Fold change	p value	Fold change	p value	Fold change	p value	Fold change	p value
Adm	Mm00437438_g1	0,73	0,9943	1,04	0,9998	1,21	0,8842	1,74	0,0547
Angpt2	Mm00545822_m1	0,98	0,7515	1,20	0,5732	1,6	0,0018	1,4	0,0503
Arg1	Mm00475991_m1	0,9	1,0000	0,6	0,5254	0,7	0,6659	0,8	0,8602
Ccl2	Mm00441242_m1	0,8	0,7564	1	0,7564	1,2	0,0069	1,3	0,8181
Ccl5	Mm01302428_m1	0,6	0,7280	0,7	0,1434	1,3	0,1456	1,2	0,5205
Ccl7	Mm01308393_g1	1,1	0,2307	1,1	0,7320	1,3	0,0764	1,4	0,0401
CXCL1	Mm04207460_m1	0,8	0,0001	1	1,0000	1	1,0000	1	0,9996
Cxcl11	Mm00444662_m1	0,8	0,0000	0,6	0,0010	0,9	0,6513	1,2	0,2332
Cxcl12	Mn00445553_m1	0,7	0,0005	1	0,0269	1,1	0,9990	1,5	0,8806
Cxcl9	Mn00434946_m1	0,7	0,0701	0,3	1,0000	0,8	0,9697	0,7	0,0235
Cxcr4	Mm01292123_m1	0,9	0,1719	0,8	0,5534	1,2	0,5746	0,8	0,3763
Dpp4	Mm00494548_m1	0,9	0,7411	1,3	0,4409	1,7	0,0035	1,3	0,2601
Foxp3	Mm00475162_m1	0,5	0,0461	0,7	0,0721	1,3	0,1546	1,1	0,9804
Il12b	Mm00434174_m1	0,7	0,0317	1	0,9978	1,5	0,0387	1,2	0,5766
Il6	Mm01210733_m1	0,7	1,0000	0,8	0,5132	1,1	0,8366	0,7	0,3231
Lgals1	Mm00839408_g1	1,1	0,0276	1,1	0,4676	1,1	0,4529	1,1	0,1457
Lox	Mm00495386_m1	0,8	0,9171	1	1,0000	1,3	0,4322	1,6	0,0591
Mrc1/CD206	Mm00485172_m1	0,7	0,0065	0,8	0,1696	0,8	0,1370	0,7	0,0049
Nos2	Mm00440502_m1	0,7	0,9985	1	0,9999	1,4	0,6653	2,7	0,0006
Sema3b	Mm00436477_m1	0,9	0,9055	1,2	0,7213	1,4	0,1947	2	0,0005
Sema3c	Mm00443121_m1	0,9	0,9965	1	0,9998	0,9	0,9902	1,1	0,9164
Slc2a1 /Glut1	Mm00441480_m1	0,96	0,9999	1,32	0,9999	1,08	0,9999	1,59	0,0342
Stc1	Mm01322191_m1	0,7	0,4492	0,9	0,9633	1,5	0,0060	1,1	0,7461
Stc2	Mm00441560_m1	0,9	0,1580	1,2	0,4341	1,5	0,0060	1,9	0,0000
Tgfb1	Mm00441729_g1	0,8	0,0000	1,2	0,0377	1,5	0,0000	1,3	0,0005
Thsb1	Mm00449031_m1	0,7	0,9972	1	0,9990	1,2	0,4935	1	1,0000
Vegfa	Mm00437306_m1	0,8	0,9489	1,3	0,6749	1,4	0,0387	1,4	0,0574
Vegfc	Mm00437311_m1	0,8	0,000003	1	0,9992	1,2	0,0720	0,9	0,8473
