# Supplementary material for: Tasquinimod triggers an early change in the polarization of tumor associated macrophages in the tumor microenvironment
Source: J Immunother Cancer. 2015 Dec 15;3:53. doi: 10.1186/s40425-015-0098-5 (PMC4678646; doi:10.1186/s40425-015-0098-5)
Supplement: Additional file 9: Table S3. — Gene expression profiling in CD11b+ macrophages derived from tumors treated with tasquinimod after 1 and 3 days of exposure using Taqman technology (Life Technolgies). The results were presented as fold change of treated tumors to control group levels. (PDF 106 kb) [file 40425_2015_98_MOESM9_ESM.pdf]

Table S3

Fold changes in gene expression in CD11b+ after 1 day and 3 days of exposure

Mouse Genes	Assay Reference	Fold change 1 day	P	Fold change 3 days	P
Angpt2	Mm00545822_m1	**0.6**	0.1317	**0.7**	0.4602
Arg1	Mm00475991_m1	**0.8**	0.8915	**0.7**	0.6471
Bsg	Mm01144228_g1	**0.9**	0.8702	**0.9**	0.8961
Ccl2	Mm00441242_m1	**1.2**	0.4385	**1.1**	0.7806
Ccl5	Mm01302428_m1	**1.1**	0.9777	**0.9**	0.8634
Ccl7	Mm01308393_g1	**1.3**	0.5206	**1.2**	0.7577
Cxcl11	Mm00444662_m1	**1.6**	0.2929	**1.6**	0.3481
Cxcl12	Mn00445553_m1	**0.2**	0.0058	**0.1**	0.0041
Cxcl2	Mm01193538_m1	**0.6**	0.3027	**0.8**	0.6279
Cxcl9	Mn00434946_m1	**1.3**	0.6917	**1.3**	0.7807
Cxcr4	Mm01292123_m1	**0.8**	0.4929	**0.7**	0.2077
Cxcr7	Mm00432610_m1	**0.3**	0.0054	**0.3**	0.0041
Fgf2	Mm00433287_m1	**0.5**	0.0006	**0.3**	0.0002
Fn1	Mm01256734_m1	**0.6**	0.0399	**0.6**	0.0578
Gpnmb	Mm01328586_g1	**1.1**	0.9434	**1.6**	0.1690
Ifng	Mm01168134_m1	**0.6**	0.0606	**0.4**	0.0143
Il10	Mn00439614_m1	**0.5**	0.0398	**0.6**	0.1134
Il12b	Mm00434174_m1	**1.4**	0.3698	**1.2**	0.7905
Il6	Mm01210733_m1	**0.3**	0.0136	**0.3**	0.0168
Irak1	Mm01193538_m1	**1**	0.6295	**0.9**	0.1422
Lgals1	Mm00839408_g1	**0.7**	0.0182	**0.8**	0.0279
Mrc1	Mm00485172_m1	**0.8**	0.3564	**0.8**	0.2248
Nos2	Mm00440502_m1	**1.6**	0.6943	**2.6**	0.1895
Nrp1	Mm00435379_m1	**0.7**	0.0299	**0.7**	0.0289
Ager	Mm01134790_g1	**1**	0.9547	**1**	0.9766
Socs-1	Mm01342740_g1	**1.4**	0.2037	**1.4**	0.2797
Tgfb1	Mm00441729_g1	**1.1**	0.4687	**1**	0.8477
Tlr4	Mm00445273_m1	**1**	0.9475	**0.9**	0.5324
Tnf	Mm00443258_m1	**0.8**	0.1785	**0.9**	0.3169
Thbs1	Mm00449031_m1	**0.5**	0.1220	**0.5**	0.0856
Vegfa	Mm00437306_m1	**0.8**	0.6283	**0.8**	0.6253
Vegfc	Mm00437311_m1	**0.3**	0.0487	**0.1**	0.0487
Ppia	Mm03302254_g1				
Hmbs	Mm01143545_m1				
