# Supplementary material for: Tasquinimod triggers an early change in the polarization of tumor associated macrophages in the tumor microenvironment
Source: J Immunother Cancer. 2015 Dec 15;3:53. doi: 10.1186/s40425-015-0098-5 (PMC4678646; doi:10.1186/s40425-015-0098-5)
Supplement: Additional file 10: Table S4. — Fold change in protein expression levels of treated tumors compared to control group after 1, 3, 5 and 7 days of exposure using Luminex Technology. (p < 0.05; One way ANOVA). (PDF 44 kb) [file 40425_2015_98_MOESM10_ESM.pdf]

Table S4.

Fold change in protein expression in tumors after 1, 3 , 5 and 7 days of exposure

	1 Day		3 Days		5 Days		7 Days	
	Fold change	p	Fold change	p	Fold change	p	Fold change	p
IP-10	0,35	0,34	0,48	0,83	1,3	0,43	1,2	0,70
G-CSF	0,14	0,30	1,2	0,23	1,2	0,49	2,2	0,14
MIP-2	0,50	0,68	2,4	0,23	ND		ND	
MIG	0,25	0,36	0,44	0,92	1,5	0,37	0,73	0,54
IL1-alpha	0,53	0,66	0,86	0,63	ND		ND	
IL-6	2,7	0,37	2,0	0,49	1,3	0,47	1,0	0,98
IL-10	0,78	0,53	1,4	0,48	0,48	0,47	0,16	0,18
IL-12(p40)	1,0	0,56	1,2	0,93	2,1	0,023	4,8	0,011
IL-17	0,74	0,98	1,1	0,62	ND		ND	
KC	0,71	0,40	1,1	0,68	1,6	0,21	1,6	0,18
MCP-1	0,46	0,41	1,2	0,38	1,2	0,44	1,3	0,51
MIP-1beta	0,25	0,65	2,3	0,54	ND		ND	
RANTES	0,33	0,20	0,47	0,44	0,53	0,49	0,56	0,50

ND NOT DETECTABLE
