# Supplementary material for: Tasquinimod triggers an early change in the polarization of tumor associated macrophages in the tumor microenvironment
Source: J Immunother Cancer. 2015 Dec 15;3:53. doi: 10.1186/s40425-015-0098-5 (PMC4678646; doi:10.1186/s40425-015-0098-5)
Supplement: Additional file 11: Table S5. — (A) Summary of tested kinases in different cellular assays. The respective cell line, ligand, ligand concentration, and duration of stimulation with the ligand are indicated. (B) Cellular IC50 values for indicated compounds on the inhibition of EGF-R, FGF-R2, IGF1-R, KIT, MET, SRC, TIE 2, VEGF-R2 and VEGF-R3 kinase activity. Each compound was tested in 8 different concentrations ranging from 33.0 × 10−11 to 1.0 × 10^−5^ (in duplicate). (PDF 70 kb) [file 40425_2015_98_MOESM11_ESM.pdf]

Table S5.

A.

Target kinase	Cell line	Ligand	Concentration	Duration of stimulation
**EGF-R**	A431	EGF	50 ng/ml	3 min
**FGF-R2**	Kato-III	no stimulation		
**IGF1-R**	MEF	IGF-1	100 ng/ml	3 min
**KIT**	M07e	SCF	100 ng/ml	3 min
**MET**	MKN45	no stimulation		
**SRC**	MEF	no stimulation		
**TIE2**	CHO	Na-Orthovanadate	10 mM	15 min
**VEGF-R2**	HUE	VEGF-A	100 ng/ml	3 min
**VEGF-R3**	MEF	no stimulation		

B.

	**IC50 (M)**			
**Compound**	**EGF-R**	**FGF-R2**	**IGF1-R**	**KIT**
Tasquinimod	>1,05E-05	>1,05E-05	>1,05E-05	>1,05E-05
Lapatinib	1,70E-08	-	-	-
Vargatef	-	1,90E-07	-	-
staurosporine	-	-	2,40E-06	-
Sunitinib	-	-	-	4,10E-10

	**IC50 (M)**		
**Compound**	**MET**	**SRC**	**TIE2**
Tasquinimod	>1,05E-05	>1,05E-05	>1,05E-05
PHA665752	3,70E-08	-	-
Dasatinib	-	4,00E-08	-
Sorafenib	-	-	6,30E-07

	**IC50 (M)**	
**Compound**	**VEGF-R2**	**VEGF-R3**
Tasquinimod	>1,05E-05	>1,05E-05
Sunitinib	2,20E-09	8,90E-09
