# Supplementary material for: Tasquinimod triggers an early change in the polarization of tumor associated macrophages in the tumor microenvironment
Source: J Immunother Cancer. 2015 Dec 15;3:53. doi: 10.1186/s40425-015-0098-5 (PMC4678646; doi:10.1186/s40425-015-0098-5)
Supplement: Additional file 12: Table S6. — List of probes used for mRNA quantification using Syber Green technology. (PDF 26 kb) [file 40425_2015_98_MOESM12_ESM.pdf]

# Table S6

**Primers used for target genes**

| Gene | Primer sequence 5’ to 3’ |
|---|---|
| β-Actin fw<br>rew | 5’- ATG CTC CCC GGG CTG TAT-3’,<br>5´-CAT AGG AGT CCT TCT GAC CCA TTC -3’ |
| Ywhaz fw<br>rew | AAC AGC TTT CGA TGA AGC CAT,<br>TGG GTA TCC GAT GTC CAC AAT |
| CD206 fw<br>(Mrc1) rew | GCA AAT GGA GCC GTC TGT GC,<br>CTC GTG GAT CTC CGT GAC AC |
| Arg-1 fw<br>rew | GTG AAG AAC CCA CGG TCT GT,<br>CTG GTT GTC AGG GGA GTG TT |
| iNos fw<br>(Nos2) rew | TGG TGG TGA CAA GCA CAT TT,<br>AAG GCC AAA CAC AGC ATA CC |
| Cxcl9 fw<br>rew | TCA ACA AAA GAG CTG CCA AA,<br>GCA GAG GCC AGA AGA GAG AA |
| Cxcl11 fw<br>rew | TCC TTT CCC CAA ATA TCA CG,<br>CAG CCA TCC CTA CCA TTC AT |
| Il-6 fw<br>rew | CCG GAG AGG AGA CTT CAC AGA G,<br>ATC TGC CAA TGC ATG AAG TCC GTG |
| Il-12β fw<br>rew | GTG TGG ATC CAA AGC AAT AC,<br>GTC TGC TCA TTC ATG ACA AG |
